# Tuning the Morphology of Spray-Coated Biohybrid Beta-lactoglobulin:TiBALDh Films with pH for Water-Based and Nanostructured Titania

**DOI:** 10.1021/jacsau.5c00097

**Published:** 2025-03-19

**Authors:** Julian E. Heger, Julija Reitenbach, Lucas P. Kreuzer, Guangjiu Pan, Ting Tian, Linus F. Huber, Nian Li, Benedikt Sochor, Matthias Schwartzkopf, Stephan V. Roth, Alexandros Koutsioubas, Peter Müller-Buschbaum

**Affiliations:** † Department of Physics, Chair for Functional Materials, TUM School of Natural Sciences, Technical University of Munich, James-Franck-Str. 1, Garching 85748 Germany; ‡ School of Physics, University of Electronic Science and Technology of China, Chengdu 610106, China; § Advanced Light Source, Lawrence Berkeley National Laboratory, 6 Cyclotron Rd, Berkeley, California 94720, United States; ∥ Deutsches Elektronen-Synchrotron DESY, Notkestraße 85, Hamburg 22607, Germany; ⊥ Department of Fibre and Polymer Technology, KTH Royal Institute of Technology, Teknikringen 56-58, Stockholm 114 28, Sweden; # Jülich Centre for Neutron Science (JCNS) at Heinz Maier-Leibnitz Zentrum (MLZ), Forschungszentrum Jülich GmbH, Lichtenbergstraße 1, Garching 85748, Germany

**Keywords:** biotemplating, green chemistry, grazing-incidence scattering, biohybrid morphology, nanostructured titania

## Abstract

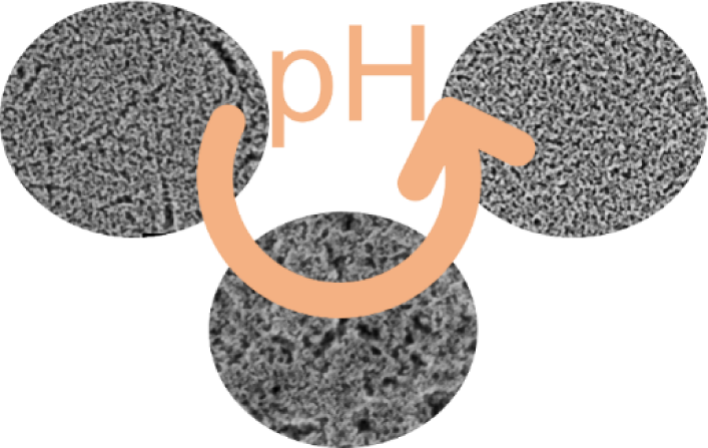

The whey protein beta-lactoglobulin (β-lg) is used as a biotemplate for the water-based synthesis of nanostructured and foam-like titania films based on its variation in supramolecular structure when denatured at different pH values. Acting as a matrix, β-lg is mixed with the water-soluble titania precursor Ti(IV) bis(ammonium lactate)dihydroxide (TiBALDh) to promote biotemplated titania precipitation. Since TiBALDh is in chemical equilibrium with anatase titania nanoparticles and Ti(IV)-lactate complexes, and this equilibrium shifts with varying pH, the influence of the pH value on the final film morphology becomes essential. This work investigates this influence for three pH values: pH 7, pH 5, i.e., close to the isoelectric point of β-lg, and pH 2. Spray coating, a method of industrial relevance, is used to fabricate biohybrid β-lg:TiBALDh foam-like films. The obtained films are calcined to combust biotemplate β-lg and achieve nanostructured titania films. To understand the influence of pH on the film morphology, grazing-incidence small-angle and wide-angle X-ray scattering (GISAXS/GIWAXS) and grazing-incidence small-angle neutron scattering (GISANS), in combination with scanning electron microscopy (SEM), are applied to both the biohybrid and biotemplated titania films. With these techniques, information about domain sizes, porosity, and crystal structure is obtained with high statistical significance. Fourier-transform infrared spectroscopy (FTIR) probes the interaction of TiBALDh and β-lg on the molecular level as a function of pH. The results underline pH as a suitable tool for tuning the morphology in biotemplated titania films.

## Introduction

Nanostructured titania (TiO_2_) has versatile potential to contribute to environmental sustainability, as this abundant and low-toxicity material has a wide range of applications, especially in the field of energy materials. For instance, nanostructured titania is used as a photocatalyst to decompose CO_2_ and produce hydrogen by water-splitting, as a key component in protective and self-cleaning coatings, and as electrodes in next-generation solar cells and batteries.^1−4^ Optimizing the morphology and crystallization of nanostructured titania is crucial to achieve optimal device performance in all these applications.^5−8^ For instance, the functionality of titania in various applications is influenced by its porosity and pore size distribution since these factors directly control the available interfacial area.^9^ Sol–gel synthesis under the direction of copolymer templates has proven to be an effective strategy for precisely tailoring the morphology of nanostructured titania.^10^ Moreover, the resulting solutions and dispersions are compatible with various large-scale industrial deposition methods, such as printing and spray coating.^11,12^ To further enhance sustainability, the use of water-based biopolymers as templates for nanostructured titania is attracting attention.^13−17^

To this end, the whey protein beta-lactoglobulin (β-lg) has emerged as a promising alternative to synthetic copolymers.^18,19^ The biomatrix is built up by the supramolecular structures of heat-denatured β-lg, which sterically directs the formation of titania domains within the pores of the biomatrix.^18^ The supramolecular structure, accompanied by the biomatrix, highly depends on the pH value at which β-lg denaturation occurs, resulting in various morphologies ranging from amyloid fibrils to spherical and worm-like aggregates.^20^ There are prominent alternatives to template the structure of metal oxides and functional materials based on other biopolymers, such as polysaccharides.^21,22^ However, being a globular protein that acts as a transport medium for vitamins by binding to ligands via hydrophobic interactions, β-lg is an interesting candidate to investigate for its templating properties on the nano and molecular levels.^23,24^ Furthermore, proteins have rich chemistry and structure due to their unique primary sequence of amino acids. The food industry successfully uses β-lg as a foaming agent, making it an ideal candidate for the aqueous synthesis of foam-like titania films.^25−27^ Certainly, nature has a rich variety of different proteins with similar properties. For instance, plant-based protein alternatives, such as pea and soy proteins, are attracting increasing attention from the nanoscience community with respect to their structural properties in applications.^28^ Building on our previous work, we have chosen β-lg as a foaming agent, as it has already been successfully applied to tailor titania nanostructures and is known to change its supramolecular structure in a controlled fashion with pH.

Furthermore, the water-based titania precursor Ti(IV) bis(ammonium lactate)dihydroxide (TiBALDh) is used in the biomineralization of titania due to its biocompatibility, where Ti(IV) ammonium lactate complexes improve the water solubility of titania.^29^ The precursor, in an aqueous solution, exists in equilibrium with anatase-TiO_2_, facilitating the precipitation of crystalline titania at room temperature, as is described by Expression (1):^30,31^
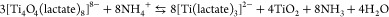
1

Besides the concentration and ionic strength, the chemical equilibrium depends on the pH value, which influences the amount of water-soluble Ti(IV) complexes. Hence, the pH value becomes essential for the water-based synthesis of nanostructured titania fabricated from β-lg and TiBALDh.

In this study, the film morphology of foam-like biohybrid β-lg:TiBALDh films, which are deposited by spray coating, is investigated at varying pH values to reveal the role of β-lg as a template for titania nanostructures. Here, spray coating aligns with economic interests as it is a deposition method with low material usage and the potential for scaling up to industrial levels.^32,33^ The surface morphology of the pristine and biotemplated titania films is investigated with scanning electron microscopy (SEM). Information about the surface is extended to the bulk morphology of the biotemplated titania films with grazing-incidence small-angle and wide-angle X-ray scattering (GISAXS/GIWAXS), where domain sizes, porosity, and crystallinity are extracted with high statistical significance and compared to the biohybrid β-lg:TiBALDh and pure β-lg films. The results are complemented by grazing-incidence small-angle neutron scattering (GISANS), which provides a different contrast sensitivity than GISAXS. The influence of pH on the domain sizes, size distribution, and crystallinity in the foam-like biohybrid films and the nanostructured, foam-like titania films after biopolymer removal is revealed. The molecular interaction between TiBALDh and β-lg is probed with Fourier-transform infrared spectroscopy (FTIR). Consequently, the results contribute to the understanding of nanostructured, foam-like, biotemplated titania films, which address ecological and economic interests and promote sustainable material synthesis relevant to energy research.

## Result and Discussion

We use spray coating to fabricate a series of four different types of films: (1) pure β-lg films, (2) foam-like β-lg:TiBALDh biohybrid composite films, (3) biotemplated titania films obtained by calcination of the biohybrid films to remove β-lg, and (4) pristine titania films prepared by calcination of the pure TiBALDh films. Each sample type is prepared under otherwise constant conditions at three different pH values, ranging from neutral (pH 7), mildly acidic (pH 5, approximating the isoelectric point of β-lg), to strongly acidic (pH 2). At these values, heat denaturation of β-lg is known to introduce worm-like structures, spherical agglomerates, and amyloid fibrils, respectively.^20^ More details on sample preparation and characterization are given in the Supporting Information.

### Influence of the pH Value on the Foam-Like Nanostructure

**Figure 1:**
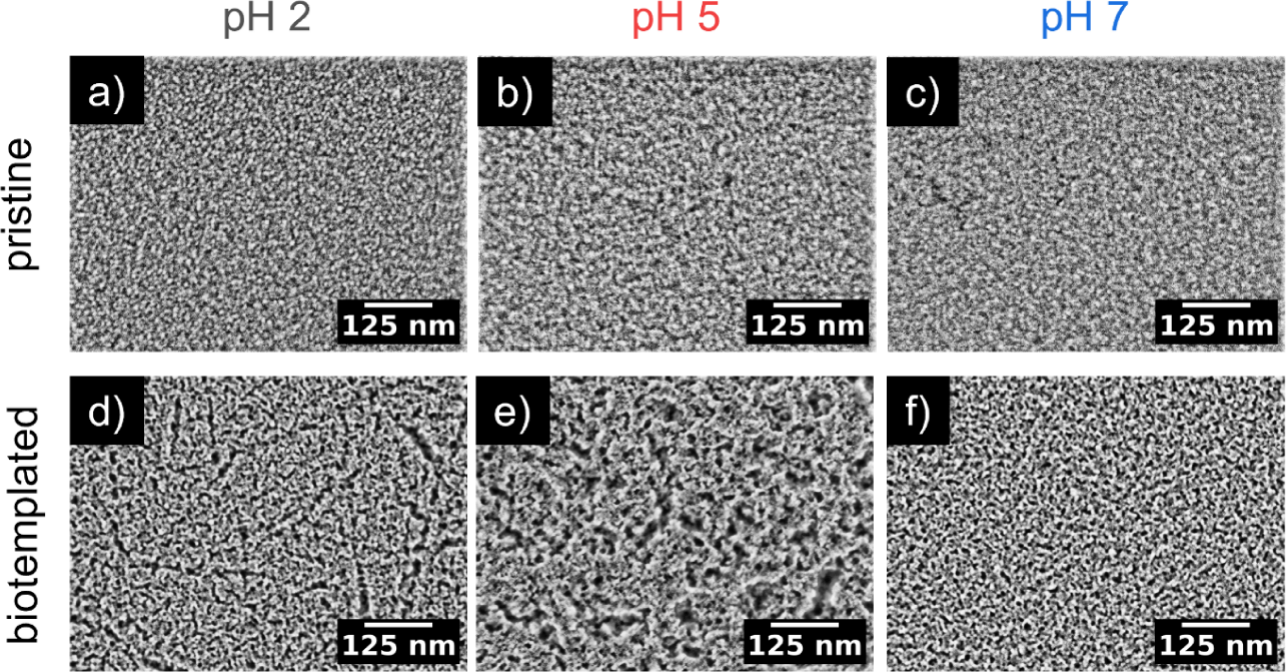
SEM images of the surface morphology of spray-coated titania films: (a–c) Surface of the pristine titania films after calcination for the three different pH values. (d–f) Surface of the biotemplated titania films after calcination for the three different pH values.

To investigate the effect of pH value on surface morphology, we conducted SEM measurements on spray-coated biotemplated titania films (Figure 1). Pristine titania films were used as a reference and compared to the biotemplated ones. The pristine titania surface exhibits a dense morphology composed of nanometer-sized particles (Figures 1a–c and S1a–c). Increasing the pH values does not result in obvious surface morphology changes. In contrast, the biotemplated foam-like titania films exhibit interconnected network surfaces composed of larger titania domains (Figures 1d–f and S1d–f). The underlying porous structure has a worm-like shape that is of comparable size at different pH values but is embedded in a changing hierarchical structure. At acidic pH 2, where β-lg forms amyloid fibrils, the hierarchical structure is formed by a fibrillar network of micrometer-elongated pores (Figures 1d and S1d). Close to the isoelectric point of β-lg at pH 5, the structure is assembled by spherical pores with larger voids having a diameter of about 100 nm (Figures 1e and S1e). At pH 7, the film shows the highest uniformity (Figures 1f and S1f). The SEM images in Figure 1 show the samples at a higher magnification than the SEM images in Figure S1, making the underlying porous structure more visible. At lower magnification, as shown in Figure S1, the hierarchical structures become more apparent. These hierarchical titania structures at pH 5 and pH 2 might enhance light scattering, thus improving absorption in photocatalytic applications. By comparing the surface morphology of the biotemplated titania to the pristine counterpart, we find that the pH-dependent influence of β-lg as a foaming agent on the resulting titania nanostructure becomes apparent.

**Figure 2:**
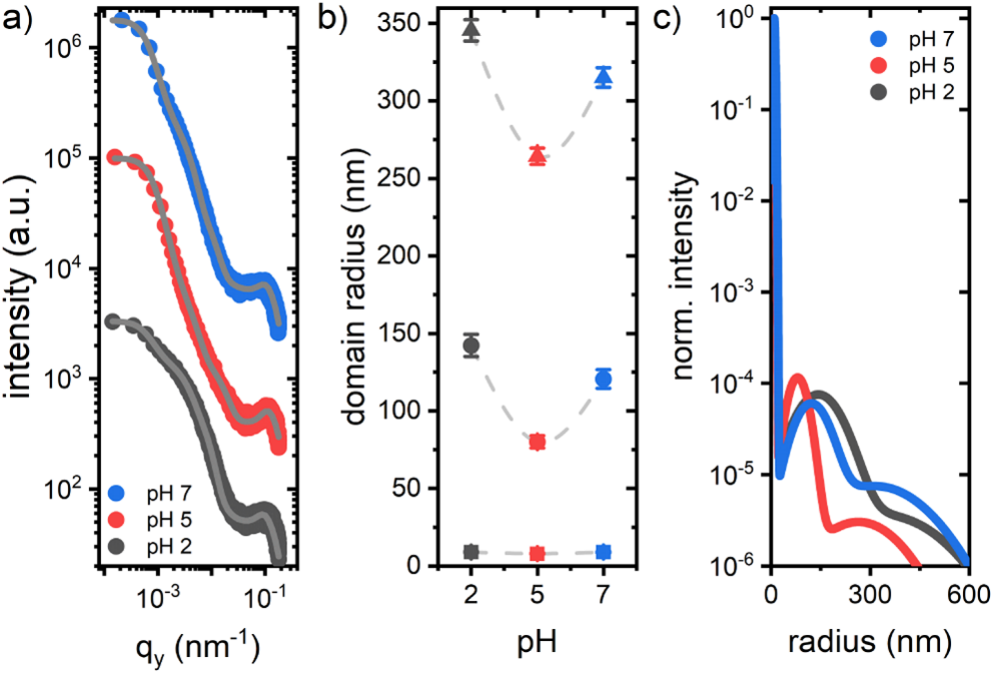
GISAXS results from biohybrid foam-like films: (a) Material characteristic horizontal line cuts from the 2D GISAXS data of biohybrid films synthesized at three different pH values. Gray lines show the corresponding model calculations, from which the domain radii are obtained. Curves are vertically shifted for the sake of clarity. (b) Domain radii as a function of pH, categorized into small (square), medium (circle), and large domains (triangle). The dashed lines are guides for the eye. (c) Normalized size distributions of the domains in the biohybrid films at different pH values.

To statistically quantify size distributions and extend the morphological information from surface to bulk, we performed GISAXS and GISANS on the β-lg:TiBALDh biohybrid films, and GISAXS on the biotemplated titania films, pure β-lg foam-like films, and pristine titania films. Figure 2 shows the GISAXS results and the obtained domain sizes for the biohybrid samples before calcination. To extract quantitative values, material-characteristic horizontal 1D intensity line cuts taken from the 2D GISAXS data were modeled with cylindrical form factors distributed on a 1D paracrystal (Figure 2a). More information about the applied model is provided in the Supporting Information and Figure S2a. From this model, the average domain sizes (Figure 2b) and their distributions (Figure 2c) were extracted. All of the data show a trimodal size distribution, which is composed of small, medium, and large domains. The values for the respective mean domain radii extracted from the model are shown in Table 1. The sizes of the small domains remain remarkably consistent across the range of pH values, whereas the medium and large domains show strong dependence on pH, with a minimum radius at pH 5. Small domains can likely be attributed to subunits, which hierarchically assemble into larger structures. We suggest that the reason for the observed difference in pH sensitivity of the respective domain types is that the nucleation of the small domains happens fast and is kinetically controlled, whereas the subsequent assembly processes leading to the larger hierarchical structures are more susceptible to pH variations.^34^ The respective standard deviations from the mean domain radii are presented in Table S1. While the distribution widths are constant for small and large domains, the medium distributions are narrowest at pH 5 and widest at pH 7. Figure S3 shows the GISAXS results from the pristine titania films as a reference to the biotemplated titania films. Domain radii and standard deviations are provided in Tables S2 and S3, respectively. The GISAXS results show with high statistical relevance that the nanostructure of the pristine titania films remains nearly unaffected by varying pH, besides a slight increase in the standard deviations of the size dispersions, and highlight the crucial influence of β-lg as a foaming agent on the nanostructure.

**Table 1:** Mean Domain Radii of the Biohybrid Size Distributions Obtained from GISAXS

radius (nm)	pH 2	pH 5	pH 7
small domains	9.0 ± 0.5	8.0 ± 0.4	9.0 ± 0.5
medium domains	142 ± 7	80 ± 4	121 ± 6
large domains	345 ± 7	264 ± 5	315 ± 6

**Figure 3:**
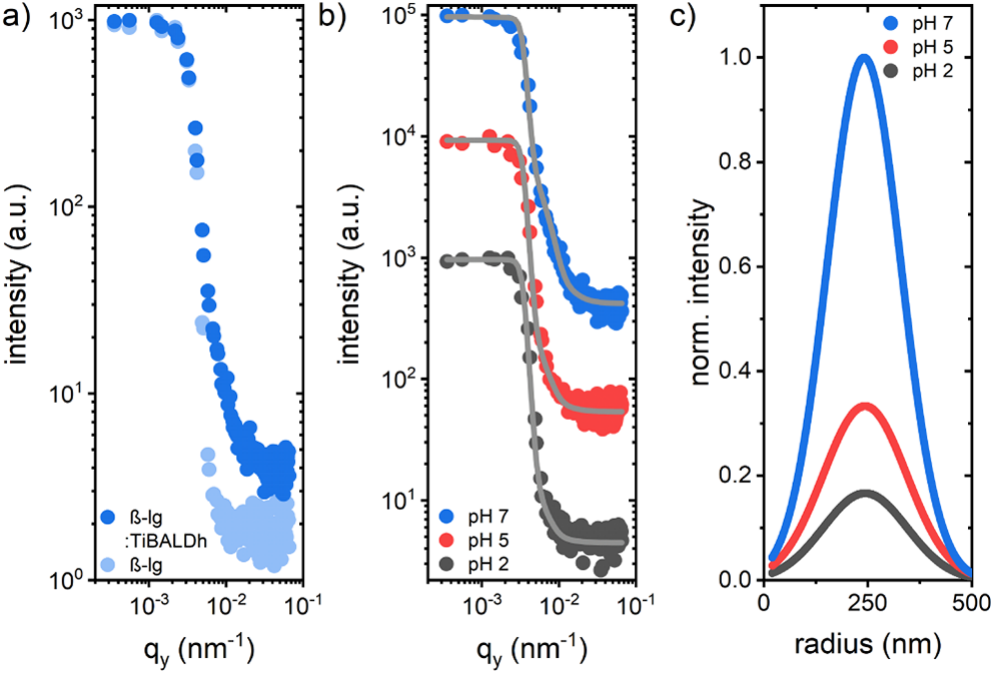
GISANS results from biohybrid foam-like films: (a) Material characteristic horizontal line cuts from the 2D GISANS data of the biohybrid film and the pure β-lg film synthesized at pH 7. (b) Material characteristic horizontal line cuts of biohybrid films synthesized at three different pH values. Gray lines show the corresponding model calculations from which the domain radii are obtained. Curves are vertically shifted for clarity. (c) Normalized size distribution of the domain radius decreases in intensity with decreasing pH value.

To extend the information on spray-coated biohybrid film morphologies, GISANS is performed in addition to GISAXS. Neutron scattering contrast differs from X-ray and hence complements morphology investigations by detecting additional domains.^35,36^ The relevant scattering length densities (SLD) are provided in Table S4. Figure 3a shows horizontal line cuts from 2D GISANS data of the biohybrid foam-like films synthesized at pH 7 and a pure β-lg reference. Here, the scattering signal from the biohybrid domains is higher. Stronger background at higher *q*_y_-values in the biohybrid films, as compared to the reference, results from incoherent scattering contributions of the hydrogen-rich TiBALDh domains. Figure 3b shows the horizontal line cuts taken from the 2D GISANS data of the biohybrid samples synthesized at three different pH values. Utilizing the same model as that for GISAXS, the GISANS domain radii are extracted. A single domain size of about 250 nm is sufficient to describe the data. With decreasing pH, the average domain size remains constant, but the overall number of domains diminishes (Figure 3c). Biohybrid domains of particularly high neutron scattering contrast are formed by interfaces between β-lg and Ti(lactate)_3_ (Figure S4, Δ(1,4), Δ(2,4), and Δ(3,4)). A diminishing intensity suggests that with decreasing pH value, a shift toward biohybrid domains with lower scattering contrast occurs, namely interfaces of β-lg and Ti_4_O_4_(lactate)_8_. This finding agrees with a shift of Expression (1) toward the right side and an increase in Ti_4_O_4_(lactate)_8_ domains with decreasing pH,^30^ thus indicating a reduced precipitation of crystalline TiO_2_ domains.

**Figure 4:**
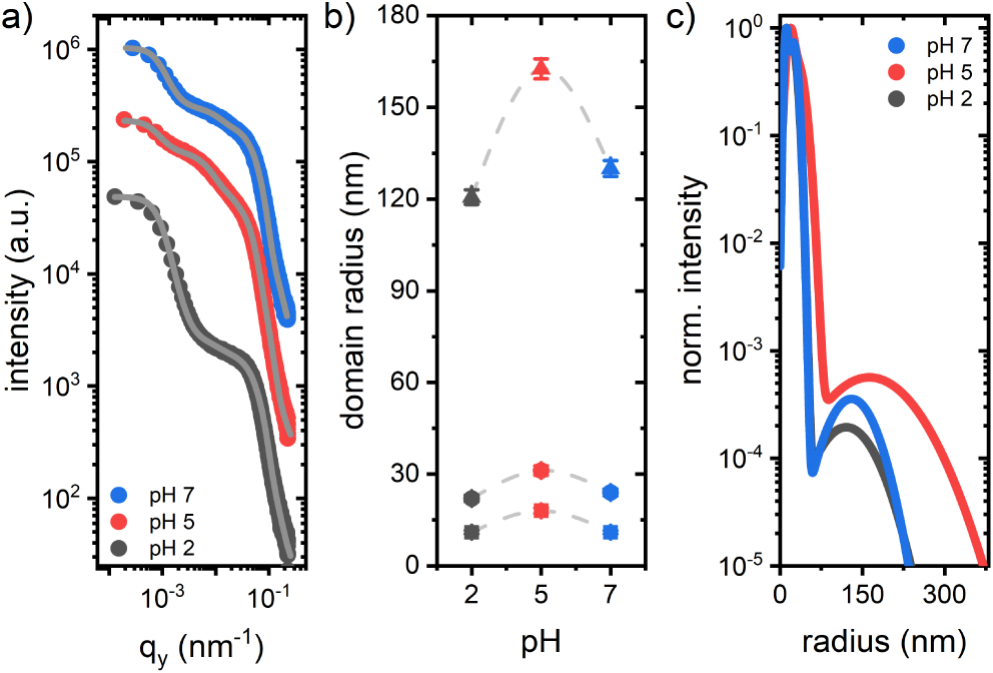
GISAXS results from nanostructured, foam-like titania films: (a) Material characteristic horizontal line cuts from the 2D GISAXS data of the nanostructured titania films after calcination at 500 °C. Gray lines show the corresponding model calculations from which the domain radii are obtained. Curves are vertically shifted for clarity. (b) Domain radii as a function of pH, categorized into small (square), medium (circle), and large domains (triangle). The dashed lines are guides to the eye. (c) Normalized size distributions of the domains in the nanostructured titania films after calcination at 500 °C.

To remove the β-lg phase from the biohybrid films and obtain nanostructured titania films, the biohybrid samples are calcined at 500 °C for 2 h. The GISAXS line cuts of the biotemplated titania films after calcination are presented in Figure 4a, from which domain sizes are extracted (Figure 4b). The values of the mean domain radii are shown in Table 2. Both pH 2 and pH 7 samples exhibit small domains with similar radii. However, the medium and large domains are slightly larger at pH 7 compared with pH 2. Notably, the largest domains for the three size categories—small, medium, and large—are observed at pH 5. This trend is opposite to that of the biohybrid films before calcination. When films before and after calcination are compared, the radii of the small domains increase, but the medium and large domains decrease. This means that upon template removal by calcination, the biohybrid composite nanostructure collapses and condenses, as the binding matrix made of β-lg leaves the compound during combustion.^37,38^ We see from the GISAXS result that the effect of domain collapse and subsequent densification is more pronounced the larger the biohybrid domain is. We suggest that the biohybrid domains contain more protein templates the larger they are, making the effect of densification more severe on larger domains than on smaller biohybrid domains. This effect is less pronounced at pH 5, as the biohybrid domains are already comparably smaller than they are at the other pH values. The small biohybrid domains, which are relatively unaffected by pH in the biohybrid sample, grew after calcination. We suggest that these domains are the subunits that hierarchically build up the medium and large biohybrid domains and undergo crystal grain growth during calcination. Furthermore, at all pH values, the distributions of the small and medium domains tend to overlap, while the large domains have the broadest size distributions (Figure 4c). The respective standard deviations from the mean domain radii are presented in Table S5.

**Table 2:** Mean Domain Radii of the Biotemplated Titania Size Distributions Obtained from GISAXS after Calcination

radius (nm)	pH 2	pH 5	pH 7
small domains	11.0 ± 0.6	18 ± 1	11.0 ± 0.6
medium domains	22 ± 1	31 ± 2	24 ± 1
large domains	121 ± 2	163 ± 3	130 ± 3

To understand the origin of the pH-induced morphological changes, it is furthermore necessary to investigate the structure of pure β-lg films (Figure S5). Comparing the β-lg:TiBALDh biohybrid with the pure β-lg films, we find that the domain sizes of the small, medium, and large structures of the pure β-lg films are comparable to the counterparts of the β-lg:TiBALDh biohybrid films, indicating that the hybrid films primarily replicate the structural template provided by β-lg (Tables S6 and S7). However, the domain sizes for both sets of films show the opposite trend with changing pH, with the strongest deviations at pH 5. This is probably due to the influence of pH on the molecular interactions between β-lg and the titania precursor TiBALDh, which slightly changes the nanostructures.

The relative degree of porosity Φ is estimated by comparing the varying scattering SLD values obtained from vertical GISAXS line cuts of the nanostructured titania after calcination (Figure S6a). The experimental SLD_exp_ and hence the electron density are taken from the Yoneda peak position at the critical angle α_c,exp_ and compared to the theoretical critical angle α_c,bulk_ = 0.19° calculated for bulk anatase titania with a material density of 3.9 g cm^–3^ and an X-ray wavelength of 1.05 Å. The porosity Φ is then estimated by Expression (2):^39^
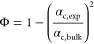
2

The biotemplated titania films have the highest porosity of 60% at pH 5, followed by 46% porosity at pH 2, and 20% porosity at pH 7 (Figure S6b). These results are in good agreement with the SEM data, in which the nanostructured titania film at pH 5 has the largest pore sizes (Figures 1d–f and S1d–f). Since a higher porosity leads to a higher surface-to-volume area, the performance of nanostructured titania in catalytic processes is typically enhanced by the greater availability of surface area at which catalytic processes take place. Besides the porosity of the achieved titania films, the crystallinity also plays an important role in catalytic performance. Compared to the changes in porosity, the changes in crystallinity shown below are minor, so we suggest that the highest porosity at pH 5 will lead to the highest catalytic performance. An experimental study, however, on the performance of biotemplated titania in applications is beyond the scope of this work.

### Influence of the pH Value on the Crystal Phase

**Figure 5:**
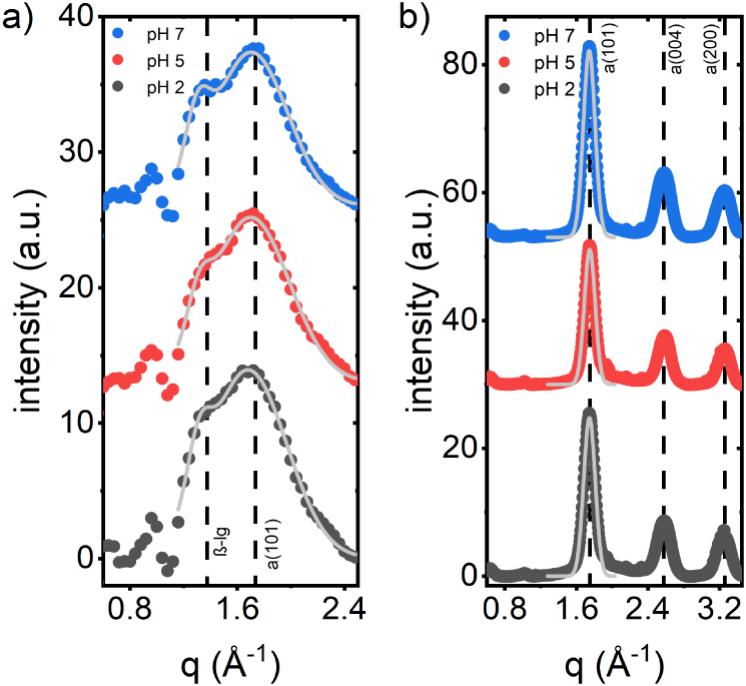
GIWAXS results on the crystal phase: *Pseudo*-XRD cuts, which give information on the crystal phase of the (a) foam-like biohybrid films and (b) nanostructured, foam-like titania films after calcination. Gray solid lines present the Gaussian fits to extract the center *q*-position and the full width at half-maximum (FWHM). All curves are vertically shifted for clarity.

Besides the nanoscale morphology, the crystal phase plays an essential role in nanostructured titania applications. To reveal the effect of the pH value, we performed GIWAXS measurements on the biohybrid films before and the nanostructured titania films after calcination. Figure 5 shows azimuthally integrated *pseudo*-X-ray diffraction (*pseudo*-XRD) cuts obtained from 2D GIWAXS data of the biohybrid films and the calcined, biotemplated titania films. A selected 2D GIWAXS image is shown in Figure S2b. The 1D cake cuts contain information about the films’ crystal phase and are analogous to powder XRD. In the biohybrid films, a fingerprint of anatase can be detected with a rather broad reflex at about *q* = 1.70 Å^–1^, which corresponds to the anatase-TiO_2_ (101) reflex. Furthermore, the reflex at about *q* = 1.30 Å^–1^ is related to β-lg and the spacing of its β-strands within a β-sheet.^40,41^ In the calcined, biotemplated titania films, the reflexes centered at about *q* = 1.75 Å^–1^, *q* = 2.58 Å^–1^, and *q* = 3.25 Å^–1^ are related to the (101), (004), and (200) reflexes of anatase-TiO_2_ (PCPDS #21–1272). More detailed information on the *q*-position and the full width at half-maximum (FWHM) of the β-sheet crystals and anatase (101) reflex in the biohybrid and foam-like titania sample, respectively, is obtained from Gaussian fits (Figure S7). The FWHM serves as a measure of the crystal coherence length, as finite crystal sizes contribute to the peak broadening of the reflexes. The shift from 1.70 Å^–1^ toward 1.75 Å^–1^ and the transition from broader toward more narrow reflexes after calcination reflect the condensation of isolated anatase-TiO_2_ nanoparticles embedded in the amorphous organic matrix into a crystalline, mesoporous titania film. Interestingly, the shift toward lower values in *q*-position and broader FWHM of the biohybrid anatase (101) reflex with decreasing pH further indicates the shift in Expression (1) toward the more amorphous Ti_4_O_4_(lactate)_8_ phase. Furthermore, the FWHM related to the biotemplated titania (101) reflex finds a minimum at pH 5, reflecting larger crystal grains within the mesoporous titania film. This observation agrees well with the GISAXS data for the biotemplated titania films, which similarly show a larger domain size at pH 5 compared with pH 2 and pH 7.

### Influence of the pH Value on the Molecular Level

**Figure 6:**
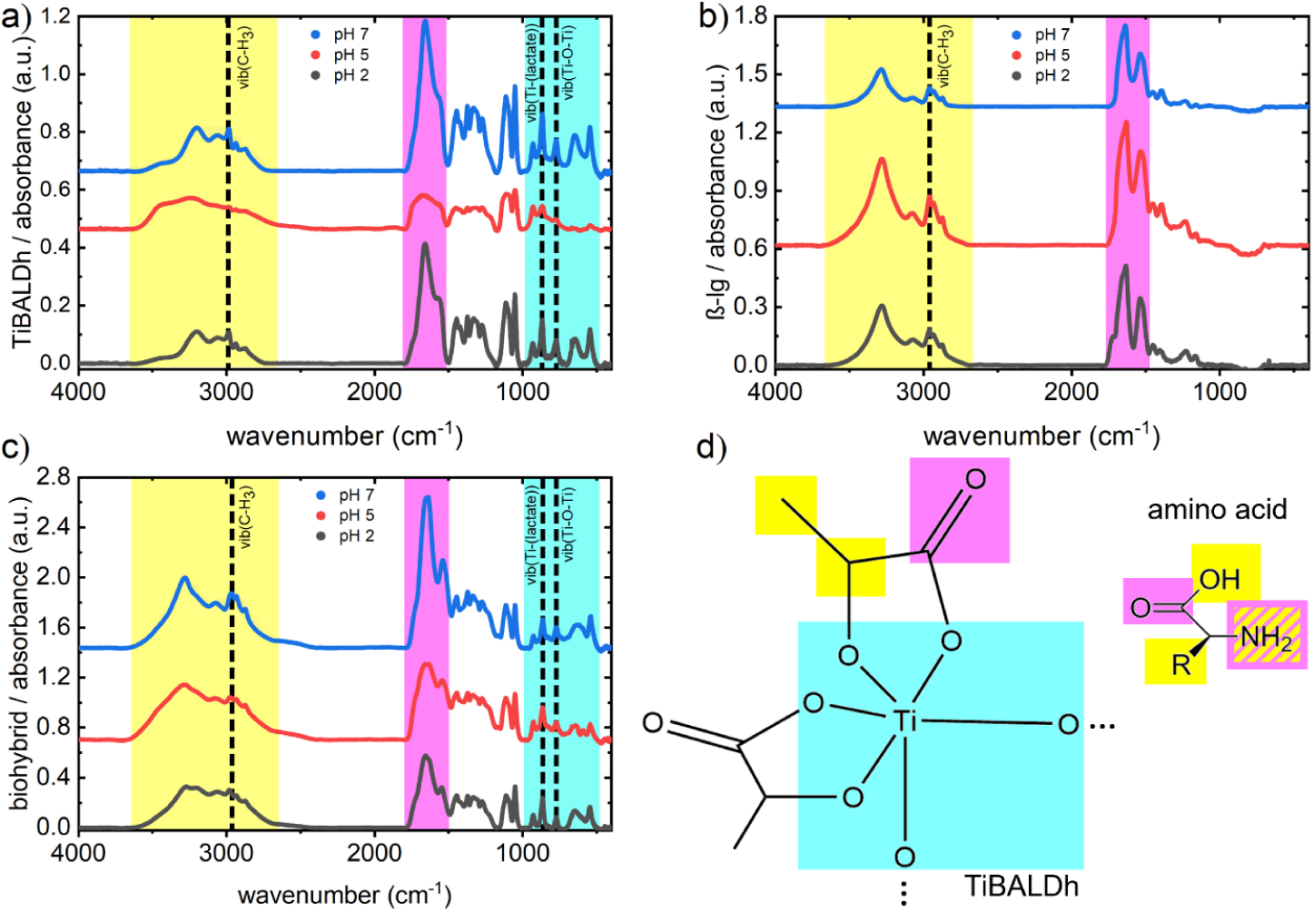
FTIR absorbance spectra: Influence of different pH values on the absorbance spectra of (a) TiBALDh films, (b) β-lg films, and (c) biohybrid films. All curves are vertically shifted for clarity. (d) Illustration of the molecular sites of TiBALDh and the amino acids in β-lg that are present in the absorbance spectra.

To analyze changes at the molecular level as a function of pH and to identify possible interactions between the biopolymer and titania precursor, we investigated drop-casted films of TiBALDh, β-lg, and the biohybrid composite with FTIR. Figure 6a shows the FTIR absorbance spectra of TiBALDh at different pH values. The spectra consist of relatively complex absorbance signals that overlap strongly in the regime from 2400 cm^–1^ to 3700 cm^–1^. Absorption peaks in this regime originate from the O–H, N–H, C–H_2_, and C–H_3_ stretching vibrations. Also, the signal arising from carbonyl groups, i.e., C=O stretching vibrations from 1500 cm^–1^ to 1800 cm^–1^, as well as the fingerprint region from 1500 cm^–1^ to 1200 cm^–1^, shows overlapping signals. Toward smaller wavenumbers, from 1000 cm^–1^ to 400 cm^–1^, the absorbance peaks become more distinct and are partially related to Ti–O vibrations. Whereas the pH 7 and pH 2 films do not show major differences besides slight intensity variations in the O–H and C=O bands, the pH 5 sample shows a strong deviation with loss in intensity and peak broadening over the entire spectra. Furthermore, peak shifts of several signals, big changes in the intensity, especially in the N–H and O–H regimes, and the development of an additional shoulder at 2625 cm^–1^ suggest the formation of different molecular interactions or even changes at a constitutional level of the precursor. The absorbance spectra of the pure β-lg samples at the respective pH values are presented in Figure 6b. The signal at about 3280 cm^–1^, which is assigned to the O–H stretching vibration, decreases in intensity with increasing pH. A smaller peak at 3072 cm^–1^ shows increasing intensity and is related to the N–H stretching. The region from 1475 cm^–1^ to 1775 cm^–1^ shows two main features. The first feature around 1600 cm^–1^ relates to the amide I band, which is characteristic of proteins that bear amide functional groups and includes overlapping signals from α-helices, β-sheets, and random coil segments.^42,43^ The second peak at about 1530 cm^–1^ is called the amide II signal, which is a contribution of several vibrations inside the amide groups. One of the main contributing vibrations is the N–H bending vibration of the amide functional group. With decreasing pH, a small shoulder evolves at 1720 cm^–1^, which indicates that inter- or intramolecular interactions in the protein are changed in the polar group. The contributions from both the TiBALDh and β-lg spectra are recognizable along with their respective pH dependencies in the absorbance spectra of the biohybrid composite at different pH values (Figure 6c).

**Figure 7:**
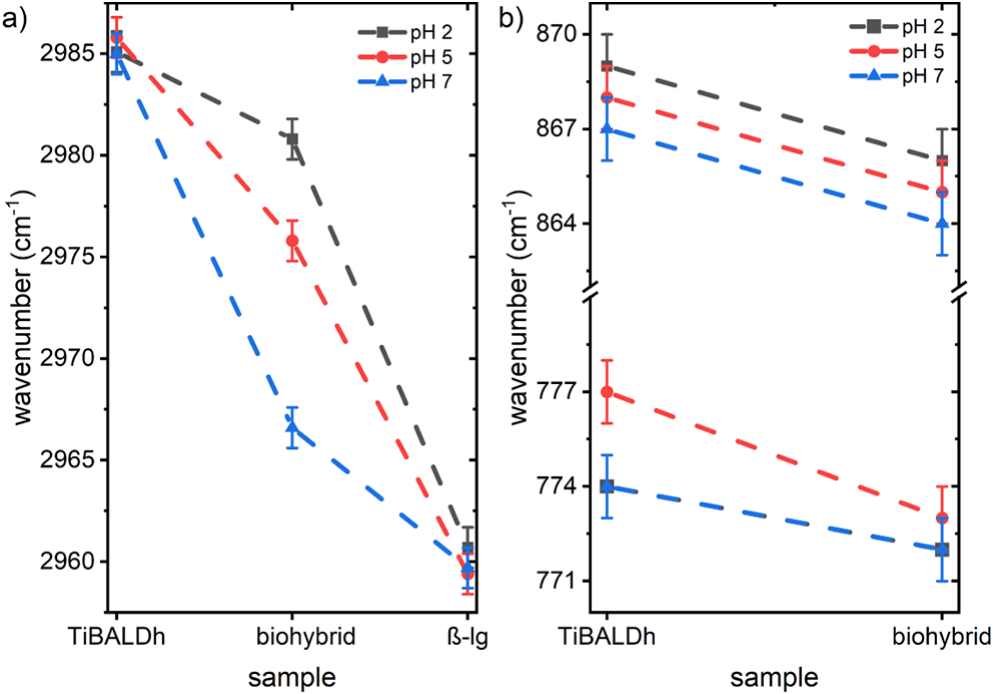
FTIR peak position shifts: (a) In contrast to the pure precursor and pure protein samples, there is a pH-dependent shift of the peak center related to the asymmetric stretching vibration of C–H at the methyl groups in the biohybrid sample. (b) Ti-(lactate) and Ti–O–Ti vibrations show a constant and lower pH dependency in the biohybrid composite, respectively, than compared to the pure precursor.

To evaluate the possible interactions in the biohybrid films more thoroughly, specific peaks are chosen and analyzed with respect to their peak center position. Figure 7a shows the absorbance peak, which originates from the asymmetric C–H stretching vibration of the methyl groups.^44^ For the pure TiBALDh samples, this peak is located at around 2985 cm^–1^ and does not change with varying pH. In the pure β-lg samples, the peak originates from methyl groups in residuals of amino acids and is located at around 2960 cm^–1^. Moreover, this β-lg peak position does not shift with varying pH. Interestingly, in the biohybrid composite sample, the signal shifts to higher wavenumbers, from about 2967 cm^–1^ at pH 7 to about 2976 cm^–1^ at pH 5 and to about 2981 cm^–1^ at pH 2. Figure 7b shows the absorbance peaks stemming from the Ti-(lactate) vibrations at around 868 cm^–1^ and the Ti–O–Ti vibrations at around 775 cm^–1^.^44,45^ In the pure TiBALDh samples, the Ti-(lactate) vibrations slightly shift toward higher wavenumbers, from 867 cm^–1^ at pH 7 to 869 cm^–1^ at pH 2. In the biohybrid composite, the Ti-(lactate) vibrations shift to lower wavenumbers while keeping the same pH dependency. The Ti–O–Ti vibrations remain constant in the biohybrid composite for pH 7 and pH 2, while they shift at pH 5 from 777 cm^–1^ in the pure TiBALDh sample to 773 cm^–1^ in the biohybrid sample, which is close to the values at pH 7 and pH 2, making the Ti–O–Ti vibrations less affected by varying pH in the biohybrid sample than in the pure TiBALDh sample. Remarkably, the Ti–O–Ti vibrations further highlight the deviating behavior on the molecular level of TiBALDh at pH 5 compared to pH 7 and pH 2.

The quantitative analysis of the FTIR absorbance spectra results in two key observations. First, due to interactions between TiBALDh and the protein, the composite becomes pH-sensitive at the molecular level. Mixing TiBALDh with β-lg affects the involved methyl groups and introduces stronger pH dependency to the asymmetric C–H stretching, whereas Ti–O–Ti vibrations are less affected by pH in the biohybrid state compared to the pure precursor. The Ti-(lactate) vibrations do not change their relative pH dependency in the biohybrid films but shift overall to slightly lower wavenumbers, which indicates a changing environment at the biohybrid interfaces. We assume that the specific interfacial change is related to the Ti-(lactate) ligands, which slightly lower their vibrational frequency by forming larger complexes in the biohybrid composite, mediating the nanoparticle surface energy. This is also reflected in the size difference of the small domains, which are larger in the biohybrid film (Figure 2b) than in the pristine film (Figure S3b). This observation might stem from hydrophobic interactions between functional groups of the protein and the lactate ligands of TiBALDh, which are absent in the pure TiBALDh samples and increase with decreasing pH, as the globular protein changes its conformational states, thus exposing more of its buried hydrophobic regions. Second, the absorbance spectra of the pure TiBALDh and the biohybrid samples show prominent deviations at pH 5, which likely is a key factor in the deviating morphology at pH 5, as revealed by GISAXS. This observation might originate in the proximity to the isoelectric point of both β-lg and TiBALDh, in the vicinity of pH 5^31^, leading to reduced electrostatic repulsion and tighter binding of the molecules. Consequently, an increasing number of hydrogen bonds are formed, which further benefits from a pronounced formation of carboxylic acids from the ester groups upon hydrolysis.

## Conclusion

In summary, we use different aggregates of the denatured whey protein β-lg as a biotemplate and the water-soluble precursor TiBALDh to fabricate mesoporous titania films with tunable morphology via spray coating. We systematically investigated the impact of pH on the film morphology, crystal structure, and molecular level. Compared to the surface morphology of pristine titania films, the influence of β-lg as a foaming agent on the resulting titania nanostructure demonstrates a clear pH dependency. All titania films consist of crystalline titania in the anatase phase. The GISAXS and GISANS data further reveal that the biohybrid films primarily replicate the structural template provided by β-lg. Remarkably, the trend observed in the biotemplated titania films with GISAXS shows that the nanostructured titania films have the largest domain sizes for small, medium, and large structures at pH 5. This is consistent with the GIWAXS results that the FWHM related to the biotemplated anatase-TiO_2_ (101) reflex is narrower at pH 5, and the strongly deviating FTIR absorbance spectra of TiBALDh are shown at pH 5. Moreover, the biotemplated titania film at pH 5 shows the highest porosity of 60%. Therefore, the influence of pH on the morphology of foam-like biohybrid films and titania films after calcination is a critical aspect that determines the final nanostructure and porosity. The results demonstrate that controlling the supramolecular structure of β-lg by pH-adjusted heat denaturation tailors the porosity and nanostructure of spray-coated titania films when applied in a water-based sol–gel synthesis.

## Experimental Section

### Materials

The biopolymer β-lg (lyophilized powder, ≥ 90% PAGE) and the titania precursor TiBALDh (50 wt % in H_2_O) were purchased from Sigma-Aldrich, Germany. Hydrochloric acid (37%) was bought from Carl Roth, Germany. All materials were used without further purification. More information on the sample fabrication is provided in the Supporting Information.

### Methods

#### SEM

The SEM measurements were performed at a working distance of 5 mm and an accelerating voltage of 5 kV (Zeiss NVision 40).

#### GISAXS/GIWAXS

The GISAXS and GIWAXS measurements were performed at the beamline P03 MiNaXS of the storage ring PETRA III (DESY, Hamburg, Germany).^46^ The wavelength was set to 1.05 Å, corresponding to 11.8 keV photon energy, and the sample was placed 4085 mm from the GISAXS detector (Pilatus 2M, Dectris, pixel size 172 × 172 μm^2^) and 260 mm from the GIWAXS detector (LAMBDA 9M, X-Spectrum, pixel size 55 × 55 μm^2^). More information on the GISAXS/GIWAXS data analysis is provided in the Supporting Information.

#### GISANS

The GISANS measurements were performed at the instrument MARIA (Heinz Maier-Leibnitz Zentrum, MLZ, Garching, Germany), provided by the Jülich Centre for Neutron Science (JCNS).^47^ The incident angle was set to 0.7°, and the scattered neutrons with a wavelength of 10 Å were detected at a distance of 1910 mm from the sample. More information on the GISANS data analysis is provided in the Supporting Information.

#### FTIR

The FTIR samples were placed in an Equinox 55 (Bruker) FTIR system in transmission mode, which was purged with CO_2_-free and dry air. The FTIR measurements were conducted with a resolution of 2 cm^–1^ and 125 scans per measurement. Baseline correction was performed with a silicon wafer reference, and atmospheric compensation was considered.

## Data Availability

All relevant data are included in the paper and its Supporting Information. The data can also be found at the following public repository: https://doi.org/10.14459/2025mp1774331.

## Supporting Information Available

Information on sample preparation, GISAXS/GISANS and GIWAXS analysis, scattering length densities, additional SEM, GISAXS, and GIWAXS data (PDF)

## References

[ref1] Haider A. J.; Jameel Z. N.; Al-Hussaini I. H. M. Review On: Titanium Dioxide Applications. Energy Proc. 2019, 157, 17–29. 10.1016/j.egypro.2018.11.159.

[ref2] Lana G. M.; Bello I. T.; Adedokun O. M.; Adenigba V. O.; Jubu P. R.; Adedokun O.; Sanusi Y. K.; Dhlamini M. S.; Awodugba A. O. One-Dimensional TiO_2_ Nanocomposite-Based Photoanode for Dye-Sensitized Solar Cells: A Review. Sol. Energy 2024, 279, 112850 10.1016/j.solener.2024.112850.

[ref3] Pipes R.; Bhargav A.; Manthiram A. Nanostructured Anatase Titania as a Cathode Catalyst for Li-CO_2_ Batteries. ACS Appl. Mater. Interfaces 2018, 10 (43), 37119–37124. 10.1021/acsami.8b13910. 30299075

[ref4] González-Morales J.; Mosa J.; Ishiyama S.; Rosero-Navarro N. C.; Miura A.; Tadanaga K.; Aparicio M. Carbon-Free Cathode Materials Based on Titanium Compounds for Zn-Oxygen Aqueous Batteries. Batteries 2024, 10 (3), 94 10.3390/batteries10030094.

[ref5] Li N.; Guo R.; Chen W.; Körstgens V.; Heger J. E.; Liang S.; Brett C. J.; Hossain M. A.; Zheng J.; Deimel P. S.; et al. Tailoring Ordered Mesoporous Titania Films via Introducing Germanium Nanocrystals for Enhanced Electron Transfer Photoanodes for Photovoltaic Applications. Adv. Funct. Mater. 2021, 31 (34), 2102105 10.1002/adfm.202102105.

[ref6] O’Regan B.; Grätzel M. A. Low-Cost, High-Efficiency Solar Cell Based on Dye-Sensitized Colloidal TiO_2_ Films. Nature 1991, 353 (6346), 737–740. 10.1038/353737a0.

[ref7] Lewis C. S.; Li Y. R.; Wang L.; Li J.; Stach E. A.; Takeuchi K. J.; Marschilok A. C.; Takeuchi E. S.; Wong S. S. Correlating Titania Nanostructured Morphologies With Performance as Anode Materials for Lithium-Ion Batteries. ACS Sustainable Chem. Eng. 2016, 4 (12), 6299–6312. 10.1021/acssuschemeng.6b00763.

[ref8] Perlich J.; Memesa M.; Diethert A.; Metwalli E.; Wang W.; Roth S. V.; Timmann A.; Gutmann J. S.; Müller-Buschbaum P. Preservation of the Morphology of a Self-Encapsulated Thin Titania Film in a Functional Multilayer Stack: An X-Ray Scattering Study. ChemPhysChem 2009, 10 (5), 799–805. 10.1002/cphc.200800800. 19226498

[ref9] Sowards K.; Medina H. Hierarchical Enhanced Surface Area Structures and Their Associated Applications With Titania. Appl. Mater. Today 2023, 35, 101962 10.1016/j.apmt.2023.101962.

[ref10] Kim K.-W.; Park B.; Kim J.; Jo C.; Kim J. K. Recent Progress in Block Copolymer Soft-Template-Assisted Synthesis of Versatile Mesoporous Materials for Energy Storage Systems. J. Mater. Chem. A 2023, 11 (14), 7358–7386. 10.1039/D2TA09353G.

[ref11] Alanazi T. I. Current Spray-Coating Approaches to Manufacture Perovskite Solar Cells. Results Phys. 2023, 44, 106144 10.1016/j.rinp.2022.106144.

[ref12] Burkitt D.; Searle J.; Watson T. Perovskite Solar Cells in N-I-P Structure With Four Slot-Die-Coated Layers. R. Soc. Open Sci. 2018, 5 (5), 172158 10.1098/rsos.172158. 29892402 PMC5990809

[ref13] Ivanova A.; Fattakhova-Rohlfing D.; Kayaalp B. E.; Rathouský J.; Bein T. Tailoring the Morphology of Mesoporous Titania Thin Films Through Biotemplating With Nanocrystalline Cellulose. J. Am. Chem. Soc. 2014, 136 (16), 5930–5937. 10.1021/ja411292u. 24533864

[ref14] Chen Q.; Betker M.; Harder C.; Brett C. J.; Schwartzkopf M.; Ulrich N. M.; Toimil-Molares M. E.; Trautmann C.; Söderberg L. D.; Weindl C. L.; et al. Biopolymer-Templated Deposition of Ordered and Polymorph Titanium Dioxide Thin Films for Improved Surface-Enhanced Raman Scattering Sensitivity. Adv. Funct. Mater. 2022, 32 (6), 2108556 10.1002/adfm.202108556.

[ref15] Bedwell G. J.; Zhou Z.; Uchida M.; Douglas T.; Gupta A.; Prevelige P. E. Selective Biotemplated Synthesis of TiO_2_ Inside a Protein Cage. Biomacromolecules 2015, 16 (1), 214–218. 10.1021/bm501443e. 25494935

[ref16] Bawazer L. A.; Ihli J.; Levenstein M. A.; Jeuken L. J. C.; Meldrum F. C.; McMillan D. G. G. Enzymatically-Controlled Biomimetic Synthesis of Titania/Protein Hybrid Thin Films. J. Mater. Chem. B 2018, 6 (23), 3979–3988. 10.1039/C8TB00381E. 32254326

[ref17] Shopsowitz K. E.; Stahl A.; Hamad W. Y.; MacLachlan M. J. Hard Templating of Nanocrystalline Titanium Dioxide With Chiral Nematic Ordering. Angew. Chem., Int. Ed. 2012, 51 (28), 6886–6890. 10.1002/anie.201201113. 22639442

[ref18] Heger J. E.; Chen W.; Yin S.; Li N.; Körstgens V.; Brett C. J.; Ohm W.; Roth S. V.; Müller-Buschbaum P. Low-Temperature and Water-Based Biotemplating of Nanostructured Foam-Like Titania Films Using ß-Lactoglobulin. Adv. Funct. Mater. 2022, 32 (20), 2113080 10.1002/adfm.202113080.

[ref19] Bolisetty S.; Adamcik J.; Heier J.; Mezzenga R. Amyloid Directed Synthesis of Titanium Dioxide Nanowires and Their Applications in Hybrid Photovoltaic Devices. Adv. Funct. Mater. 2012, 22 (16), 3424–3428. 10.1002/adfm.201103054.

[ref20] Jung J.-M.; Savin G.; Pouzot M.; Schmitt C.; Mezzenga R. Structure of Heat-Induced β-Lactoglobulin Aggregates and Their Complexes With Sodium-Dodecyl Sulfate. Biomacromolecules 2008, 9 (9), 2477–2486. 10.1021/bm800502j. 18698816

[ref21] Boury B.; Plumejeau S. Metal Oxides and Polysaccharides: An Efficient Hybrid Association for Materials Chemistry. Green Chem. 2015, 17 (1), 72–88. 10.1039/C4GC00957F.

[ref22] Ma Y.; Morozova S. M.; Kumacheva E. From Nature-Sourced Polysaccharide Particles to Advanced Functional Materials. Adv. Mater. 2024, 36 (23), e2312707 10.1002/adma.202312707. 38391153

[ref23] Yang M. C.; Chen N. C.; Chen C.-J.; Wu C. Y.; Mao S. J. T. Evidence for Beta-lactoglobulin Involvement in Vitamin D Transport In Vivo - Role of the Gamma-Turn (Leu-Pro-Met) of Beta-lactoglobulin in Vitamin D Binding. Febs J. 2009, 276 (8), 2251–2265. 10.1111/j.1742-4658.2009.06953.x. 19298386

[ref24] Riihimäki L.; Galkin A.; Finel M.; Heikura J.; Valkonen K.; Virtanen V.; Laaksonen R.; Slotte J. P.; Vuorela P. Transport Properties of Bovine and Reindeer Beta-lactoglobulin in the Caco-2 Cell Model. Int. J. Pharm. 2008, 347 (1–2), 1–8. 10.1016/j.ijpharm.2007.06.015. 17658229

[ref25] Peng D.; Yang J.; Li J.; Tang C.; Li B. Foams Stabilized by β-Lactoglobulin Amyloid Fibrils: Effect of pH. J. Agric. Food Chem. 2017, 65 (48), 10658–10665. 10.1021/acs.jafc.7b03669. 29135243

[ref26] Moro A.; Báez G. D.; Ballerini G. A.; Busti P. A.; Delorenzi N. J. Emulsifying and Foaming Properties of β-lactoglobulin Modified by Heat Treatment. Food Res. Int. 2013, 51 (1), 1–7. 10.1016/j.foodres.2012.11.011.

[ref27] Rullier B.; Axelos M. A. V.; Langevin D.; Novales B. Beta-lactoglobulin Aggregates in Foam Films: Correlation Between Foam Films and Foaming Properties. J. Colloid Interface Sci. 2009, 336 (2), 750–755. 10.1016/j.jcis.2009.04.034. 19476951

[ref28] Zhang Y.; Dee D. R. Morphology, Formation Kinetics and Core Composition of Pea and Soy 7S and 11S Globulin Amyloid Fibrils. J. Agric. Food Chem. 2023, 71 (11), 4755–4765. 10.1021/acs.jafc.2c08704. 36890640

[ref29] Korina E.; Morozov R.; Arkhipushkin I.; Vorobiev D.; Heintz N.; Inyaev I.; Adawy A.; Mendoza R.; Vasileva I.; Dolinina T.; Avdin V.; Sozykin S.; Schelokov A.; Popov V.; Strel’tsova E.; Bol’shakov O. Surface Dehydroxylation of Nanocrystalline TiO_2_. Inorg. Chem. Commun. 2021, 126, 108478 10.1016/j.inoche.2021.108478.

[ref30] Hernández-Gordillo A.; Hernández-Arana A.; Campero-Celis A.; Vera-Robles L. I. Tibaldh as a Precursor for Biomimetic TiO_2_ Synthesis: Stability Aspects in Aqueous Media. RSC Adv. 2019, 9 (59), 34559–34566. 10.1039/C9RA05923G. 35529993 PMC9073908

[ref31] Seisenbaeva G. A.; Daniel G.; Nedelec J.-M.; Kessler V. G. Solution Equilibrium Behind the Room-Temperature Synthesis of Nanocrystalline Titanium Dioxide. Nanoscale 2013, 5 (8), 3330–3336. 10.1039/c3nr34068f. 23467564

[ref32] Maho A.; Nayak S.; Gillissen F.; Cloots R.; Rougier A. Film Deposition of Electrochromic Metal Oxides Through Spray Coating: A Descriptive Review. Coatings 2023, 13 (11), 1879 10.3390/coatings13111879.

[ref33] Roth S. V. A Deep Look into the Spray Coating Process in Real-Time-the Crucial Role of X-Rays. J. Phys.: condens. Matter 2016, 28 (40), 403003 10.1088/0953-8984/28/40/403003. 27537198

[ref34] Sui R.; Jacobs J. H.; Chou N.; Deering C. E.; Lavery C. B.; Marriott R. A. Facile Synthesis of Thermally Stable Anatase Titania with a High-Surface Area and Tailored Pore Sizes. J. Sol-Gel Sci. Technol. 2023, 107 (2), 289–301. 10.1007/s10971-023-06117-7.

[ref35] Müller-Buschbaum P. GISAXS and GISANS as Metrology Technique for Understanding the 3D Morphology of Block Copolymer Thin Films. Eur. Polym. J. 2016, 81, 470–493. 10.1016/j.eurpolymj.2016.04.007.

[ref36] Paracini N.; Gutfreund P.; Welbourn R.; Gonzalez-Martinez J. F.; Zhu K.; Miao Y.; Yepuri N.; Darwish T. A.; Garvey C.; Waldie S.; Larsson J.; Wolff M.; Cárdenas M. Structural Characterization of Nanoparticle-Supported Lipid Bilayer Arrays by Grazing Incidence X-Ray and Neutron Scattering. ACS Appl. Mater. Interfaces 2023, 15 (3), 3772–3780. 10.1021/acsami.2c18956. 36625710 PMC9880997

[ref37] Li N.; Chen W.; Song L.; Guo R.; Scheel M. A.; Yang D.; Körstgens V.; Schwartzkopf M.; Roth S. V.; Müller-Buschbaum P. In Situ Study of Order Formation in Mesoporous Titania Thin Films Templated by a Diblock Copolymer during Slot-Die Printing. ACS Appl. Mater. Interfaces 2020, 12 (51), 57627–57637. 10.1021/acsami.0c18851. 33295752

[ref38] Guldin S.; Kolle M.; Stefik M.; Langford R.; Eder D.; Wiesner U.; Steiner U. Tunable Mesoporous Bragg Reflectors Based on Block-Copolymer Self-Assembly. Adv. Mater. 2011, 23 (32), 3664–3668. 10.1002/adma.201100640. 21732558

[ref39] Wang W.; Widmann T.; Song L.; Fröschl T.; Hüsing N.; Mo G.; Wu Z.; Zhang P.; Roth S. V.; Fan H.; Müller-Buschbaum P. Aging of Low-Temperature Derived Highly Flexible Nanostructured TiO_2_ /P3HT Hybrid Films During Bending. J. Mater. Chem. A 2019, 7 (17), 10805–10814. 10.1039/C9TA01544B.

[ref40] Hofmaier M.; Heger J. E.; Lentz S.; Schwarz S.; Müller-Buschbaum P.; Scheibel T.; Fery A.; Müller M. Influence of the Sequence Motive Repeating Number on Protein Folding in Spider Silk Protein Films. Biomacromolecules 2023, 24 (12), 5707–5721. 10.1021/acs.biomac.3c00688. 37934893

[ref41] Maurer-Stroh S.; Debulpaep M.; Kuemmerer N.; Lopez de la Paz M.; Martins I. C.; Reumers J.; Morris K. L.; Copland A.; Serpell L.; Serrano L.; et al. Exploring the Sequence Determinants of Amyloid Structure Using Position-Specific Scoring Matrices. Nat. Methods 2010, 7 (3), 237–242. 10.1038/nmeth.1432. 20154676

[ref42] Barth A. Infrared Spectroscopy of Proteins. Biochim. Biophys. Acta, Protein Struct. Mol. Enzymol. 2007, 1767 (9), 1073–1101. 10.1016/j.bbabio.2007.06.004. 17692815

[ref43] Benbow N. L.; Rozenberga L.; McQuillan A. J.; Krasowska M.; Beattie D. A. ATR FTIR Study of the Interaction of TiO_2_ Nanoparticle Films with β-Lactoglobulin and Bile Salts. Langmuir 2021, 37 (45), 13278–13290. 10.1021/acs.langmuir.1c01830. 34731567

[ref44] Otieno O. V.; Csáki E.; Kéri O.; Simon L.; Lukács I. E.; Szécsényi K. M.; Szilágyi I. M. Synthesis of TiO_2_ Nanofibers by Electrospinning Using Water-Soluble Ti-Precursor. J. Therm. Anal. Calorim. 2020, 139 (1), 57–66. 10.1007/s10973-019-08398-z.

[ref45] Zeitler V. A.; Brown C. A. The Infrared Spectra of Some Ti-O-Si, Ti-O-Ti and Si-O-Si Compounds. J. Phys. Chem. 1957, 61 (9), 1174–1177. 10.1021/j150555a010.

[ref46] Buffet A.; Rothkirch A.; Döhrmann R.; Körstgens V.; Abul Kashem M. M.; Perlich J.; Herzog G.; Schwartzkopf M.; Gehrke R.; Müller-Buschbaum P.; et al. P03, the Microfocus and Nanofocus X-Ray Scattering (MiNaXS) Beamline of the PETRA III Storage Ring: The Microfocus Endstation. J. Synchrotron Radiat. 2012, 19 (4), 647–653. 10.1107/S0909049512016895. 22713902 PMC3380660

[ref47] Mattauch S.; Koutsioubas A.; Rücker U.; Korolkov D.; Fracassi V.; Daemen J.; Schmitz R.; Bussmann K.; Suxdorf F.; Wagener M.; et al. The High-Intensity Reflectometer of the Jülich Centre for Neutron Science: MARIA. J. Appl. Crystallogr. 2018, 51 (3), 646–654. 10.1107/S1600576718006994. 29896056 PMC5988004

