# Supplementary material for: Tuning the Morphology of Spray-Coated Biohybrid Beta-lactoglobulin:TiBALDh Films with pH for Water-Based and Nanostructured Titania
Source: JACS Au. 2025 Mar 19;5(4):1894–902. doi: 10.1021/jacsau.5c00097 (PMC12042038; doi:10.1021/jacsau.5c00097)
Supplement: Supplementary file 1 — au5c00097_si_001.pdf [file au5c00097_si_001.pdf]

Supporting Information

Tuning the Morphology of Spray-Coated Biohybrid Beta-lactoglobulin:TiBALDh Films with pH for Water-Based and Nanostructured Titania

Julian E. Heger[1], Julija Reitenbach[1], Lucas P. Kreuzer[1], Guangjiu Pan[1], Ting Tian[1], Linus F. Huber[1], Nian Li[2], Benedikt Sochor[3,4], Matthias Schwartzkopf[4], Stephan V. Roth[4,5], Alexandros Koutsioubas[6], Peter Müller-Buschbaum[1,*]

[1]Technical University of Munich, TUM School of Natural Sciences, Department of Physics, Chair for Functional Materials, James-Franck-Str. 1, 85748 Garching, Germany

E-mail: muellerb@ph.tum.de

[2]School of Physics, University of Electronic Science and Technology of China, Chengdu 610106, China

[3]Advanced Light Source, Lawrence Berkeley National Laboratory, 6 Cyclotron Rd, Berkeley, CA 94720, USA.

[4]Deutsches Elektronen-Synchrotron DESY, Notkestraße 85, 22607 Hamburg, Germany

[5]Department of Fibre and Polymer Technology, KTH Royal Institute of Technology, Teknikringen 56-58, 114 28 Stockholm, Sweden

[6]Jülich Centre for Neutron Science (JCNS) at Heinz Maier-Leibnitz Zentrum (MLZ), Forschungszentrum Jülich GmbH, Lichtenbergstraße 1, 85748 Garching, Germany

Keywords: biotemplating, green chemistry, grazing-incidence scattering, biohybrid morphology, nanostructured titania

Solution preparation

Solutions of 10 mg mL^{-1} ß-lactoglobulin (*ß*-lg) in deionized (DI) water were adjusted to pH 5 ± 0.5 and pH 2 ± 0.5 by the addition of 1 µL and 10 µL 12 M HCl, respectively. Without the addition of HCl, the solutions remained at pH 7 ± 0.5. Denaturation of *ß*-lg was introduced via thermal treatment at 90 °C for 5 h at pH 2 and for 30 min at pH 5 and pH 7.[1] After quenching the heated solutions in an ice bath, 2.5 mL of the respective solutions were mixed with 79.2 µL TI(IV) bis(ammonium lactate)dihydroxide (TiBALDh, 50 wt. % in DI H_2O) to obtain the biohybrid solutions. TiBALDh reference solutions were prepared by diluting the precursor with 2.5 mL DI water and the addition of 0 µL, 1 µL, or 10 µL HCl (12 M), respectively. The samples under investigation were spray-coated in a commercial spray chamber (PVA 350, Werner Wirth GmbH, Hamburg, Germany), with a spray nozzle for general purpose (781S, Nordson, USA). Pre-cleaned and surface-treated silicon substrates (p-doped, thickness = 525 ± 25 µm, 10−20 Ωcm, Si-Mat, Germany) with a dimension of 7×7 cm^2 were spray-coated by moving the spray nozzle with a speed of 150 mm s^{-1} in a grid with 5 mm spacing and a distance to the sample of 3.5 cm. The atomizing pressure of the oil-free nitrogen carrier gas was set to 0.5 bar. The samples were heated to 80 °C during deposition and annealed at 140 °C for 10 min after deposition. The wafers were used for GISANS measurements as-deposited and were cut down to about 2×2 cm^2 for GISAXS and GIWAXS measurements. FTIR samples were drop-casted from 100 µL of the respective solutions on 1×1 cm^2 silicon wafers. and dried at 140 °C for 10 min. To achieve nanostructured and crystalline titania films, the as-deposited biohybrid samples were calcined for 2 h at 500 °C with a heating ramp of 1 °C min^{-1} to remove the template *ß*-lg and the organic parts of the Ti(IV) lactate complexes. The same calcination protocol was performed for the pure TiBALDh samples as a reference. We have not applied pH conditions above pH 7, as typically acidic environments are used for sol-gel synthesis of titania nanostructures.[2,3] Given the focus of this work on the bulk structure characterization with neutrons and X-rays and the limited amount of experimental beamtime at the large-scale

research facilities, we have decided to limit our scope on the presented three pH values. These values are of particular interest, as they are lower than, close to, and higher than the isoelectric points of the involved materials, respectively. Further, *ß*-lg has its well-known supramolecular transition from worm-like to spherical to amyloid fibrils at the tested pH values of about 2, 5, and 7.[1]

Analysis of GISAXS/GISANS data

The 2D GISAXS/GISANS data was reduced to 1D intensity profiles along q_y at the critical angle $q_z(\alpha_c)$, where $\boldsymbol{q}$ is the vector of momentum transfer between incident and scattered X-rays and neutrons, with the software DPDAK.[4] The 1D intensity profiles were modeled to extract lateral domain sizes with cylindrical form factors on a 1D paracrystalline lattice within the distorted wave Born approximation, using the effective interface approximation and the local monodisperse approximation.[5,6] Within this framework, the measured scattering intensity, $I(q)$, is given as:

$$I(q) \propto \sum_k N_k \cdot \langle |F(q, R_k)|^2 \rangle \cdot \Delta_{i,j} \cdot S(q, R_k) \qquad \text{(S1)}$$

Hence, the intensity is proportional to the number of scattering centers N and the square of their electron distribution, which is the form factor $F(q)$ and relates to the size R of scattering domains k. The intensity is proportional to the scattering contrast $\Delta_{i,j}$ at the interface between two materials with a given electron density. Furthermore, the scattering intensity is also proportional to the distribution of scatterers along the sample, given by the structure factor $S(q)$. The vector q is the momentum transfer between the incoming and scattered X-ray beam. The structure factor $S(q)$ reflects a mean inter-domain distance d between domains k, and we neglected the structure factor ($S(q) = 1$) when no regular structural correlation between scattering domains was found during the modeling of the measured intensity. Vertical line cuts along q_z are taken at $q_y = 0$ to analyze the critical angle α_c and the respective film porosity of the biotemplated titania films.

Analysis of GIWAXS data

The 2D GIWAXS data was reshaped from raw detector data by transformation from pixel to ***q***-space with the software INSIGHT.[7] From the reshaped 2D GIWAXS data, 1D *pseudo*-X-ray diffraction pattern (*pseudo*-XRD) were obtained by azimuthal integration. Reflexes selected from the *pseudo*-XRD were fitted with Gaussian functions to extract their center q-position and full width at half maximum (FWHM). All information on the crystal phase has been derived from the 2D GIWAXS data and azimuthal integration to 1D pseudo-XRD cuts. In contrast to XRD, GIWAXS probes the in-plane lattice orientation and crystal phase. As seen from the Dybe-Scherrer rings in the selected 2D GIWAXS data (Figure S2b, Supporting Information), the lattice orientations are present in a powder-like isotropy, making sharp reflexes from lattice planes oriented parallel to the substrate unlikely. Hence, no further complementary XRD measurements are performed to probe the missing wedge of scattering intensity along $q_z(q_r = 0)$ of the 2D GIWAXS data.

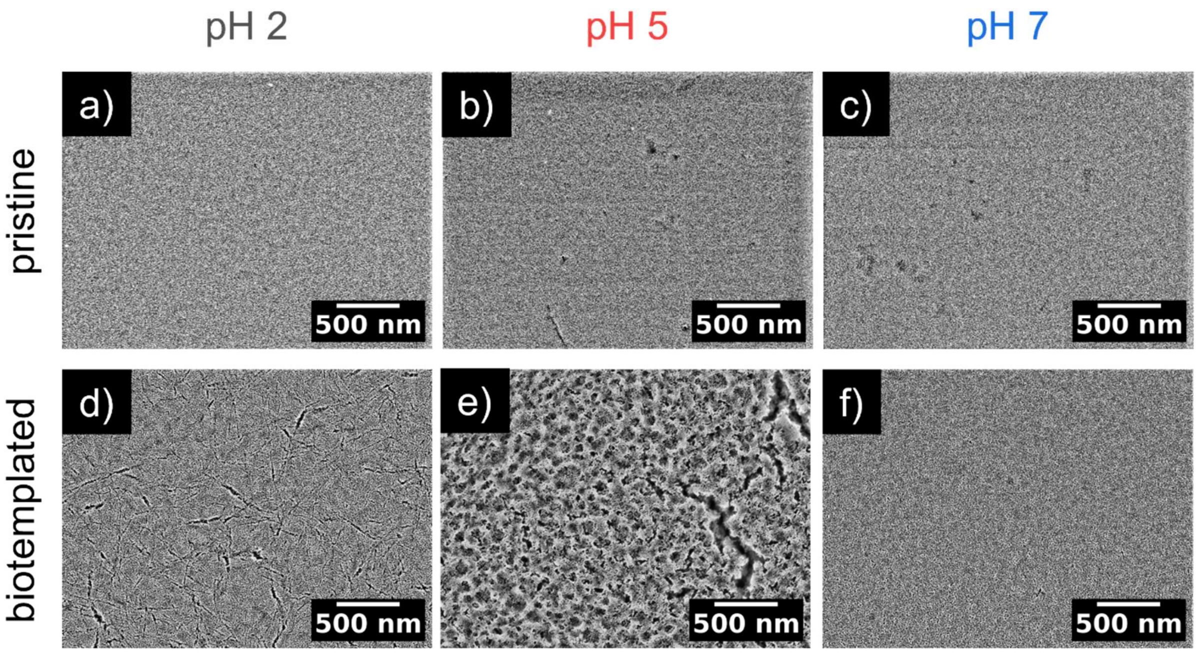

Figure S1. SEM images of the surface morphology of spray-coated titania films: a-c) Surface of the pristine titania films after calcination for the three different pH values. d-f) Surface of the biotemplated titania films after calcination for the three different pH values.

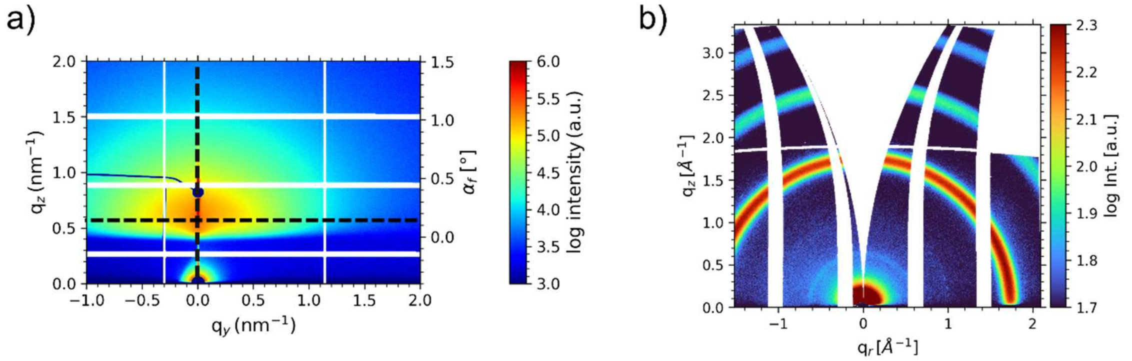

Figure S2. Selected 2D detector data. a) 2D GISAXS data with vertical and horizontal line cut positions indicated by black dashed lines. b) Reshaped 2D GIWAXS data from which *pseudo*-XRD data is obtained by azimuthal integration.

size dispersion (%)	pH 2	pH 5	pH 7
small domains	35	35	35
medium domains	40	35	45
large domains	45	45	45

Table S1. Standard deviations of the biohybrid size distributions obtained from GISAXS.

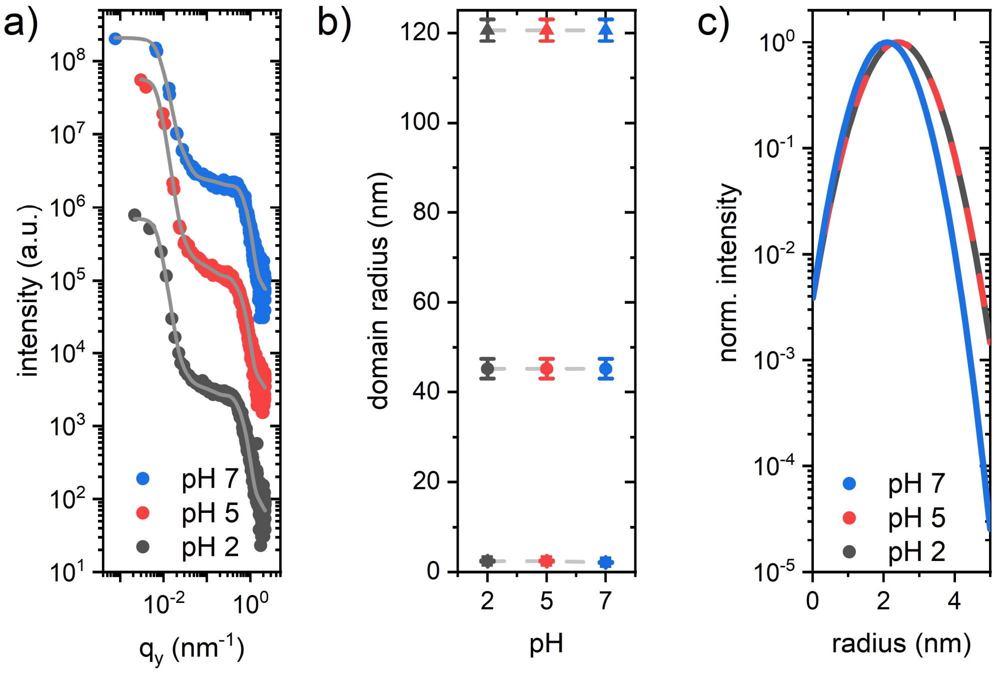

Figure S3. GISAXS results from pristine titania films: a) Material characteristic horizontal line cuts from the 2D GISAXS data of pristine titania films after calcination and synthesized at three different pH values as a reference to the biotemplated titania samples. Gray lines show the corresponding model calculations from which the domain radii are obtained. b) Domain radii as a function of pH categorized into small (square), medium (circle), and large domains (triangle). The dashed lines are guides to the eye. c) Normalized size distributions of the domains in the pristine titania films at different pH values.

radius (nm)	pH 2	pH 5	pH 7
small domains	2.4 ± 0.5	2.4 ± 0.5	2.1 ± 0.5
medium domains	45 ± 3	45 ± 3	45 ± 3
large domains	120 ± 7	120 ± 7	120 ± 7

Table S2. Mean domain radii of the pristine titania size distributions obtained from GISAXS after calcination.

size dispersion (%)	pH 2	pH 5	pH 7
small domains	30	30	30
medium domains	40	40	40
large domains	40	40	40

Table S3. Standard deviations of the pristine titania size distributions obtained from GISAXS after calcination.

Scattering length densities and scattering contrast

The X-ray and neutron scattering length densities (*SLD*s) were calculated with the NIST *SLD* calculator (https://www.ncnr.nist.gov/resources/activation/, last modified 25-March-2024) and the scattering contrast rising from the difference between the respective material *SLD* calculated via $\Delta_{i,j} = |SLD_i - SLD_j|$.

	material	X-ray SLD [10^{-6} Å^{-2}]	neutron coh. SLD [10^{-6} Å^{-2}]	neutron incoh. SLD [10^{-6} Å^{-2}]
1	$Ti_4O_4(lactate)_8$	*8.60*	*1.38*	*7.42*
2	$Ti(lactate)_3$	*8.17*	*0.62*	*1.16*
3	TiO_2	*8.66*	*1.46*	*8.26*
4	*ß-lg*	*9.09*	*1.33*	*15.8*

Table S4. Calculated X-ray and neutron scattering length densities.

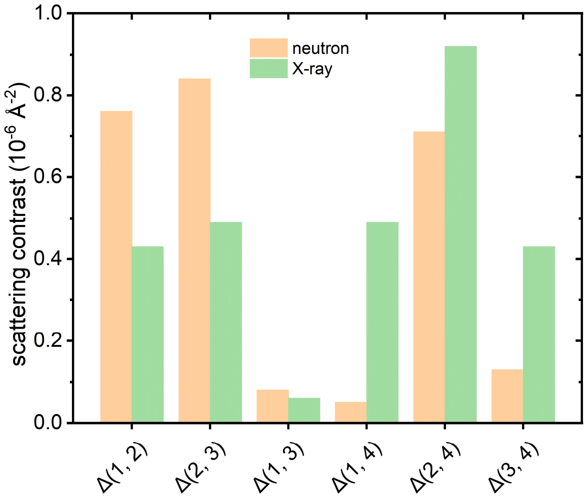

Figure S4. Contrast between X-ray and coherent neutron scattering length densities for $Ti_4O_4(lactate)_8$ abbreviated as 1, $Ti(lactate)_3$ abbreviated as 2, TiO_2 abbreviated as 3, and *ß-lg* abbreviated as 4.

**size dispersion (%)**	**pH 2**	**pH 5**	**pH 7**
small domains	35	35	30
medium domains	36	40	30
large domains	40	45	30

Table S5. Standard deviations of the biotemplated-titania size distributions obtained from GISAXS after calcination.

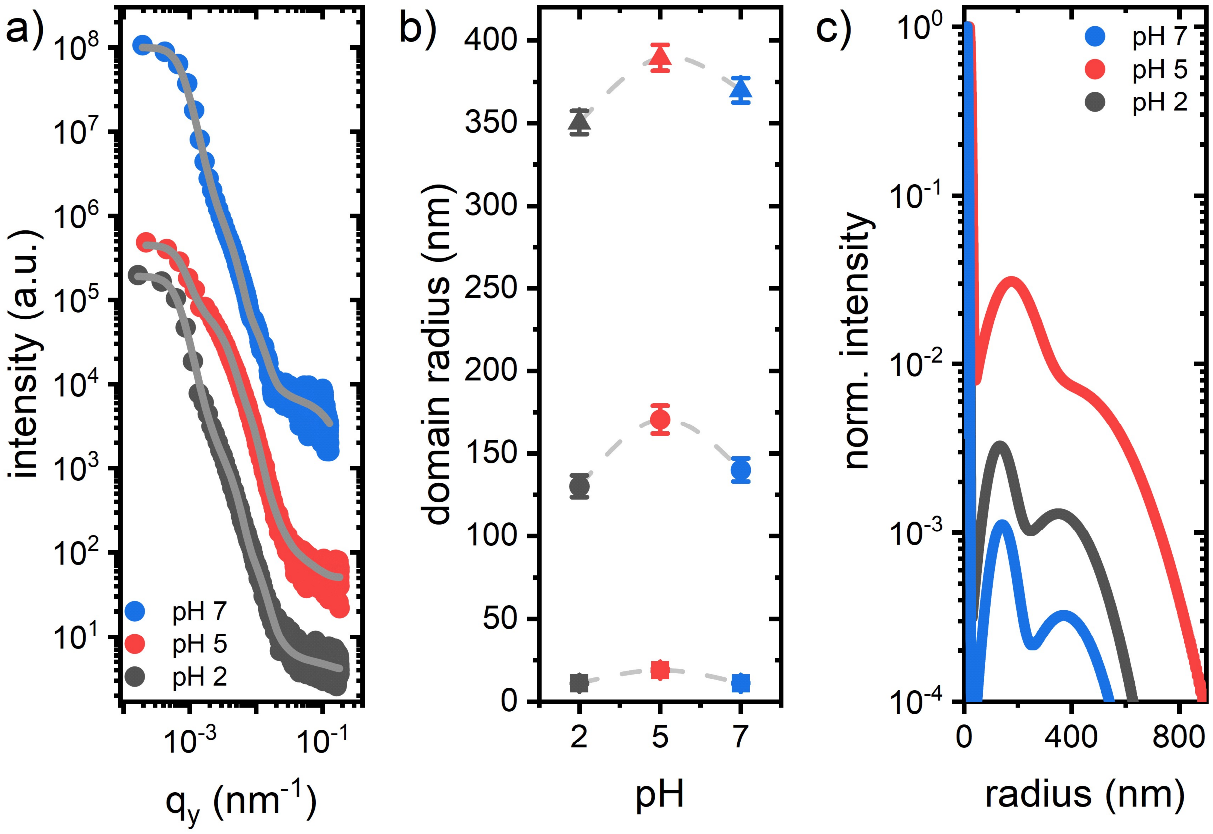

Figure S5. GISAXS results from pure *β*-lg foam-like films: a) Material characteristic horizontal line cuts from the 2D GISAXS data of *β*-lg films synthesized at three different pH values. Gray lines show the corresponding model calculations from which the domain radii are obtained. b) Domain radii as a function of pH categorized into small (square), medium (circle), and large domains (triangle). The dashed lines are guides to the eye. c) Normalized size distributions of the domains in the pure *β*-lg films at different pH values.

**radius (nm)**	**pH 2**	**pH 5**	**pH 7**
small domains	11 ± 1	19 ± 1	11 ± 1
medium domains	130 ± 7	171 ± 9	140 ± 7
large domains	350 ± 7	390 ± 8	370 ± 7

Table S6. Mean domain radii of the pure *β*-lg size distributions obtained from GISAXS.

**size dispersion (%)**	**pH 2**	**pH 5**	**pH 7**
small domains	35	30	30
medium domains	35	45	30
large domains	35	44	30

Table S7. Standard deviations of the pure *β*-lg size distributions obtained from GISAXS after calcination.

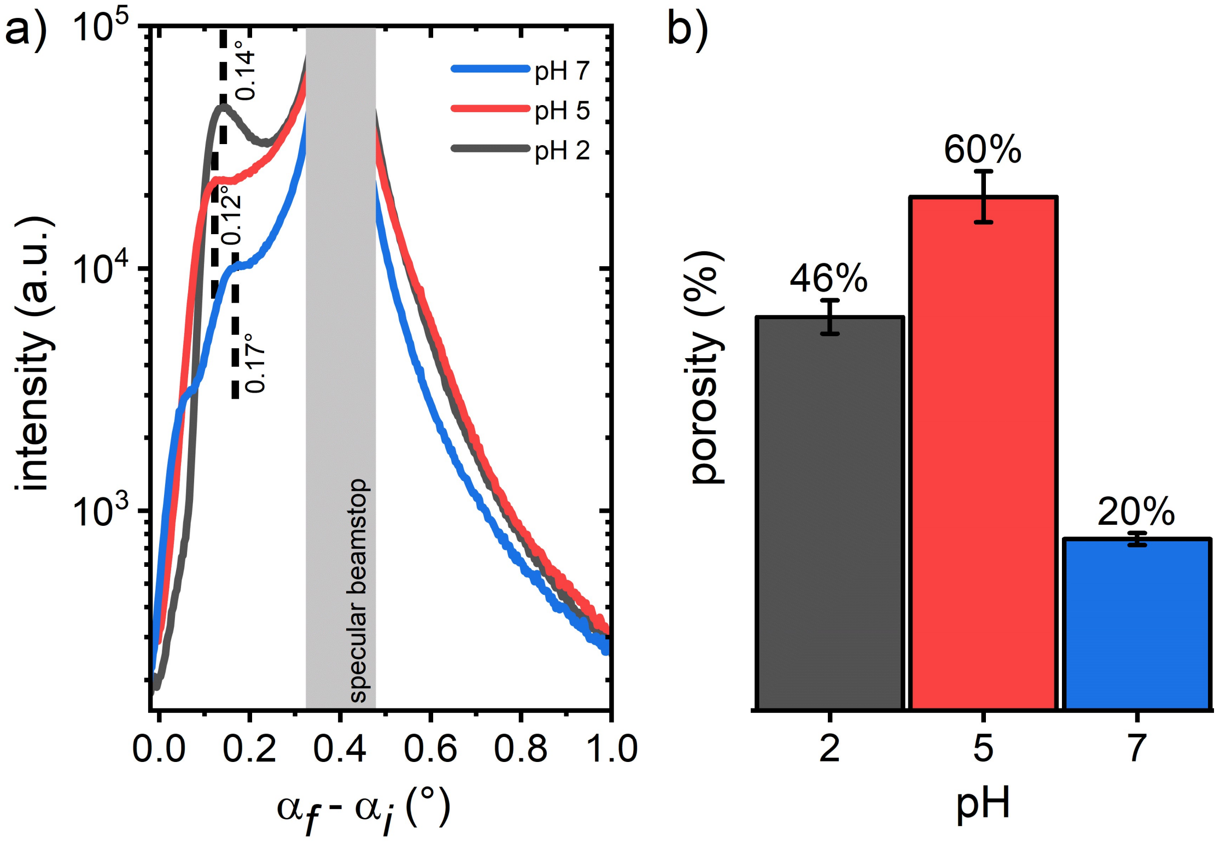

Figure S6. Vertical GISAXS line cuts of biotemplated titania as function of pH: a) Vertical line cuts show a shift in the critical angle α_c, which is related to the electron density of the calcined titania films. b) Porosity of the biotemplated films after calcination at 500 °C for the different pH, as obtained from the varying electron density.

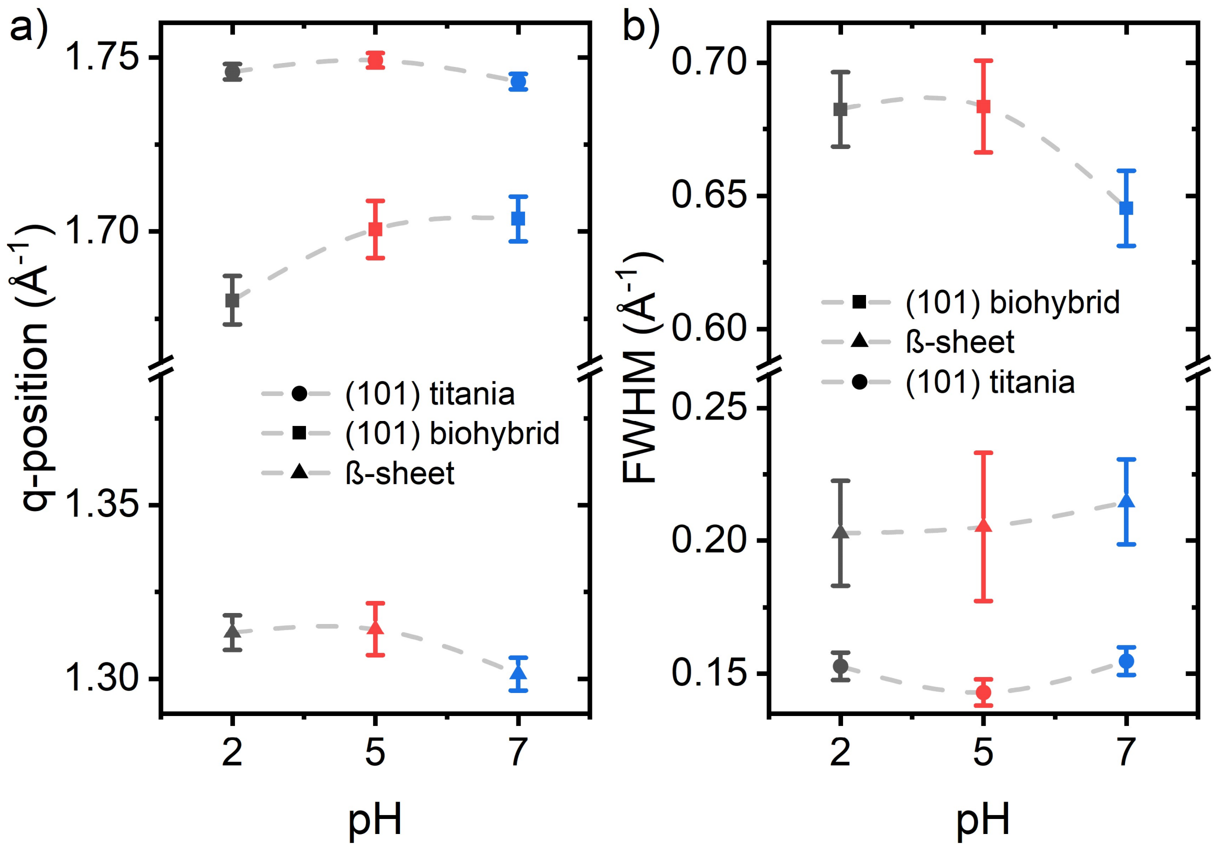

Figure S7. GIWAXS results obtained from Gaussian fits: a) *q*-position and b) FWHM of the reflexes related to the anatase (101) lattice planes in the biohybrid films as deposited and after calcination, as well as the *β*-sheets crystals of the protein.

References

(1) Jung, J.-M.; Savin, G.; Pouzot, M.; Schmitt, C.; Mezzenga, R. Structure of Heat-Induced β-Lactoglobulin Aggregates and Their Complexes With Sodium-Dodecyl Sulfate. *Biomacromolecules* **2008**, *9* (9), 2477–2486.

(2) Nikkanen, J.-P.; Kanerva, T.; Mäntylä, T. The Effect of Acidity in Low-Temperature Synthesis of Titanium Dioxide. *J. Cryst. Growth* **2007**, *304* (1), 179–183.

(3) Chang, C.; Rad, S.; Gan, L.; Li, Z.; Dai, J.; Shahab, A. Review of the Sol–Gel Method in Preparing Nano TiO_2 for Advanced Oxidation Process. *Nanotechnol. Rev.* **2023**, *12* (1).

(4) Benecke, G.; Wagermaier, W.; Li, C.; Schwartzkopf, M.; Flucke, G.; Hoerth, R.; Zizak, I.; Burghammer, M.; Metwalli, E.; Müller-Buschbaum, P.; Trebbin, M.; Förster, S.; Paris, O.; Roth, S. V.; Fratzl, P. A Customizable Software for Fast Reduction and Analysis of Large X-Ray Scattering Data Sets: Applications of the New DPDAK Package to Small-Angle X-Ray Scattering and Grazing-Incidence Small-Angle X-Ray Scattering. *J. Appl. Crystallogr.* **2014**, *47* (Pt 5), 1797–1803.

(5) Hofmaier, M.; Heger, J. E.; Lentz, S.; Schwarz, S.; Müller-Buschbaum, P.; Scheibel, T.; Fery, A.; Müller, M. Influence of the Sequence Motive Repeating Number on Protein Folding in Spider Silk Protein Films. *Biomacromolecules* **2023**, *24* (12), 5707–5721.

(6) Heger, J. E.; Chen, W.; Yin, S.; Li, N.; Körstgens, V.; Brett, C. J.; Ohm, W.; Roth, S. V.; Müller-Buschbaum, P. Low-Temperature and Water-Based Biotemplating of Nanostructured Foam-Like Titania Films Using β-Lactoglobulin. *Adv. Funct. Mater.* **2022**, *32* (20), 2113080.

(7) Reus, M. A.; Reb, L. K.; Kosbahn, D. P.; Roth, S. V.; Müller-Buschbaum, P. INSIGHT: In Situ Heuristic Tool for the Efficient Reduction of Grazing-Incidence X-ray Scattering Data. *J. Appl. Crystallogr.* **2024**, *57* (Pt 2), 509–528.
